# Supplementary figures and images for: Systems-level network modeling of Small Cell Lung Cancer subtypes identifies master regulators and destabilizers
Source: PLoS Comput Biol. 2019 Oct 31;15(10):e1007343. doi: 10.1371/journal.pcbi.1007343 (PMC6860456; doi:10.1371/journal.pcbi.1007343)

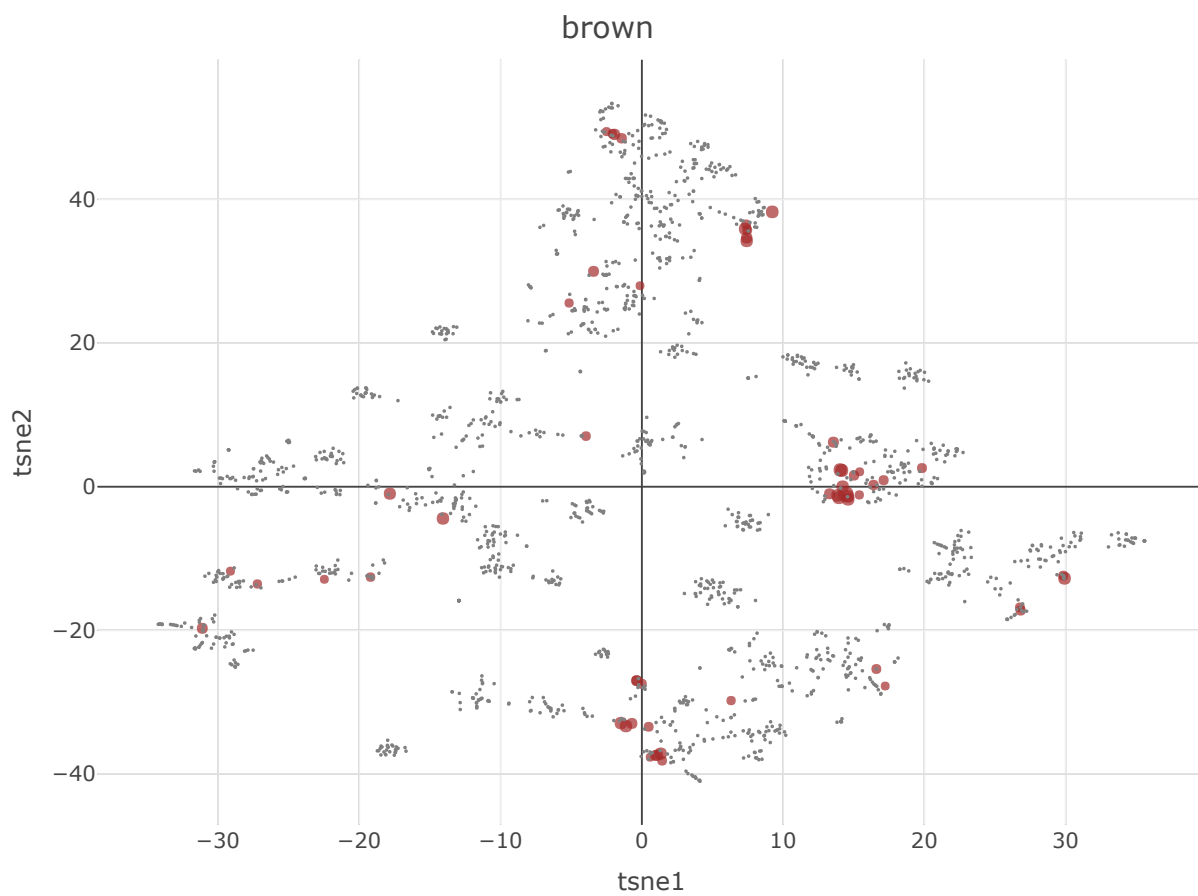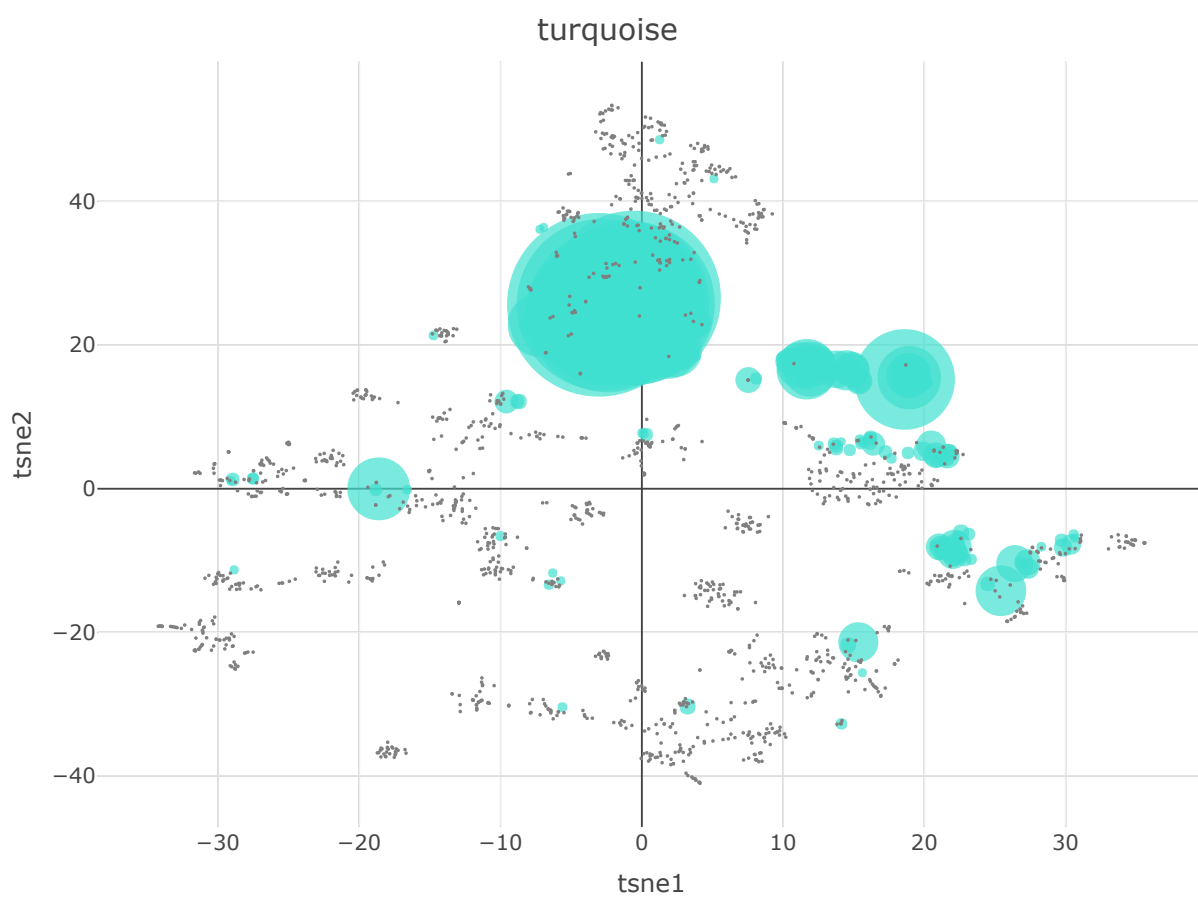

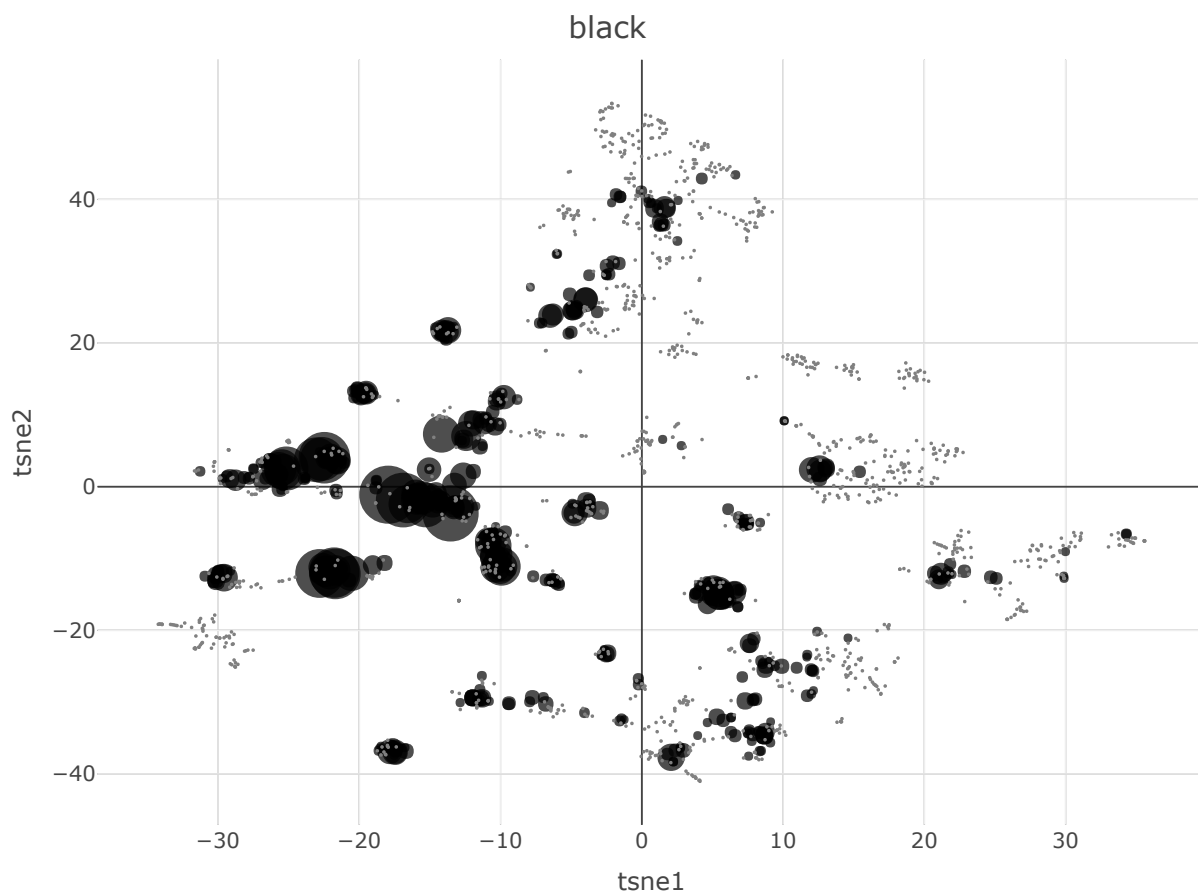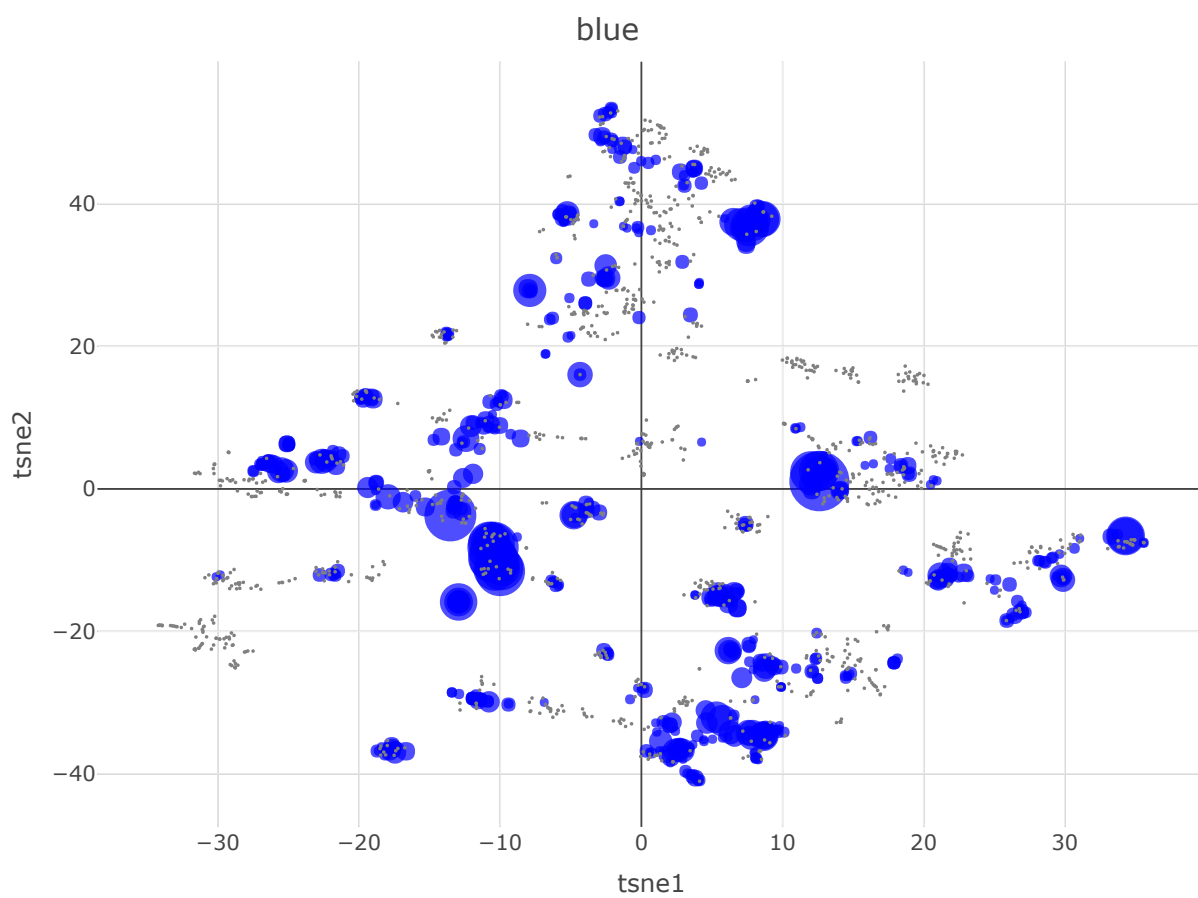

midnightblue

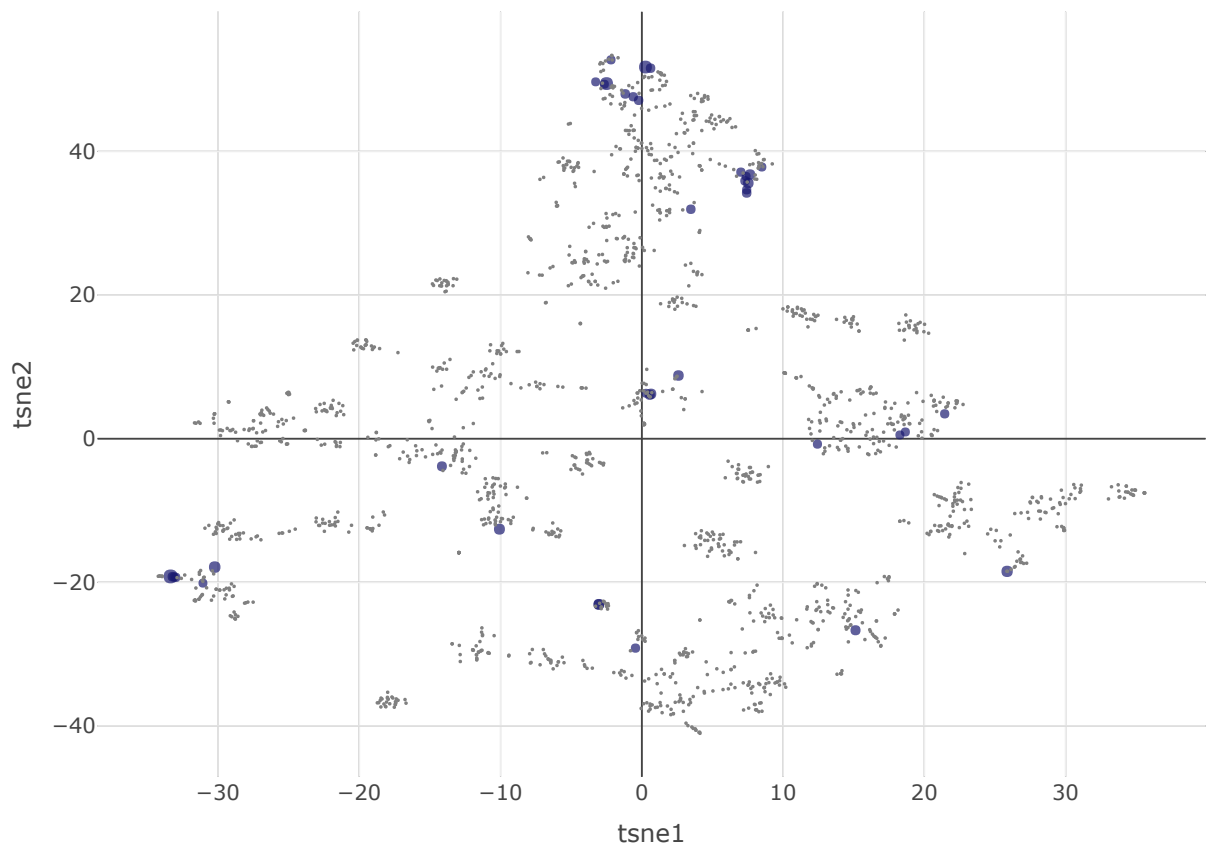

salmon

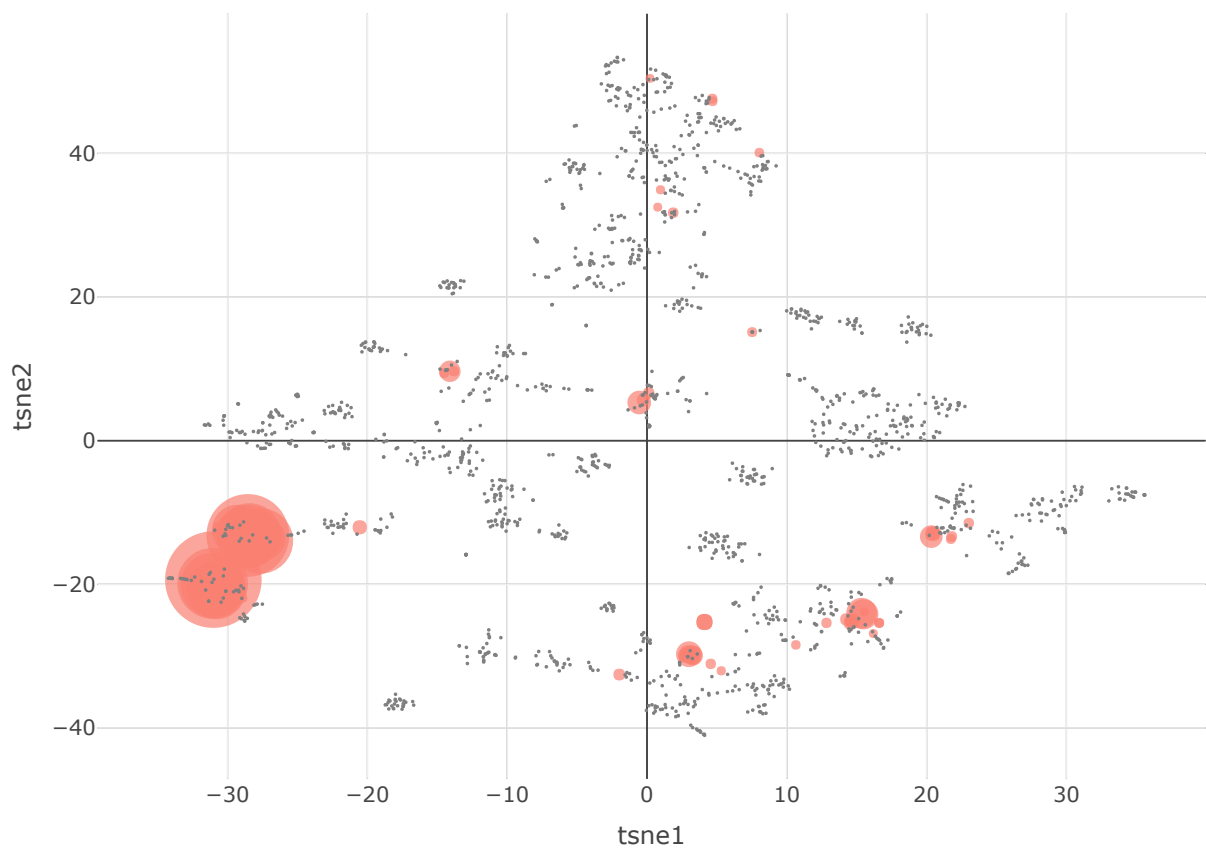

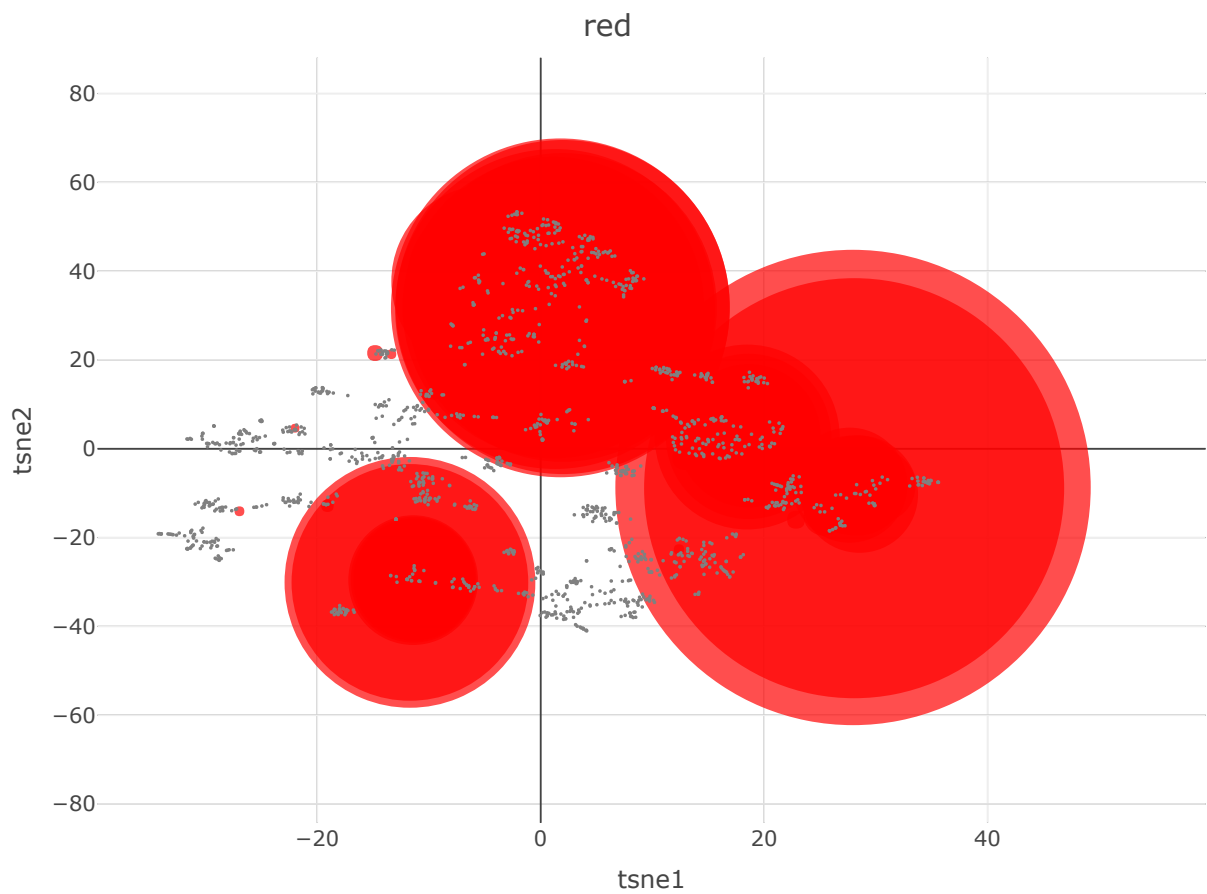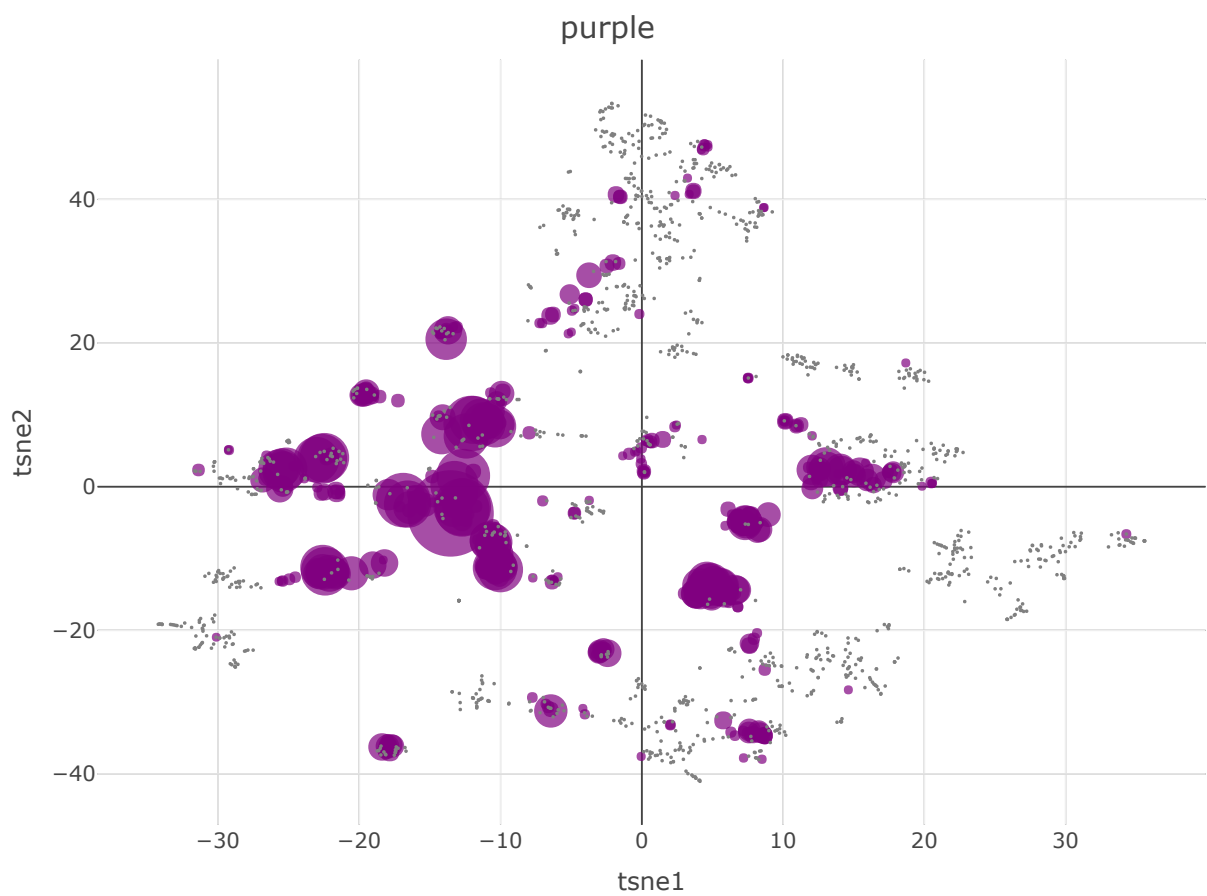

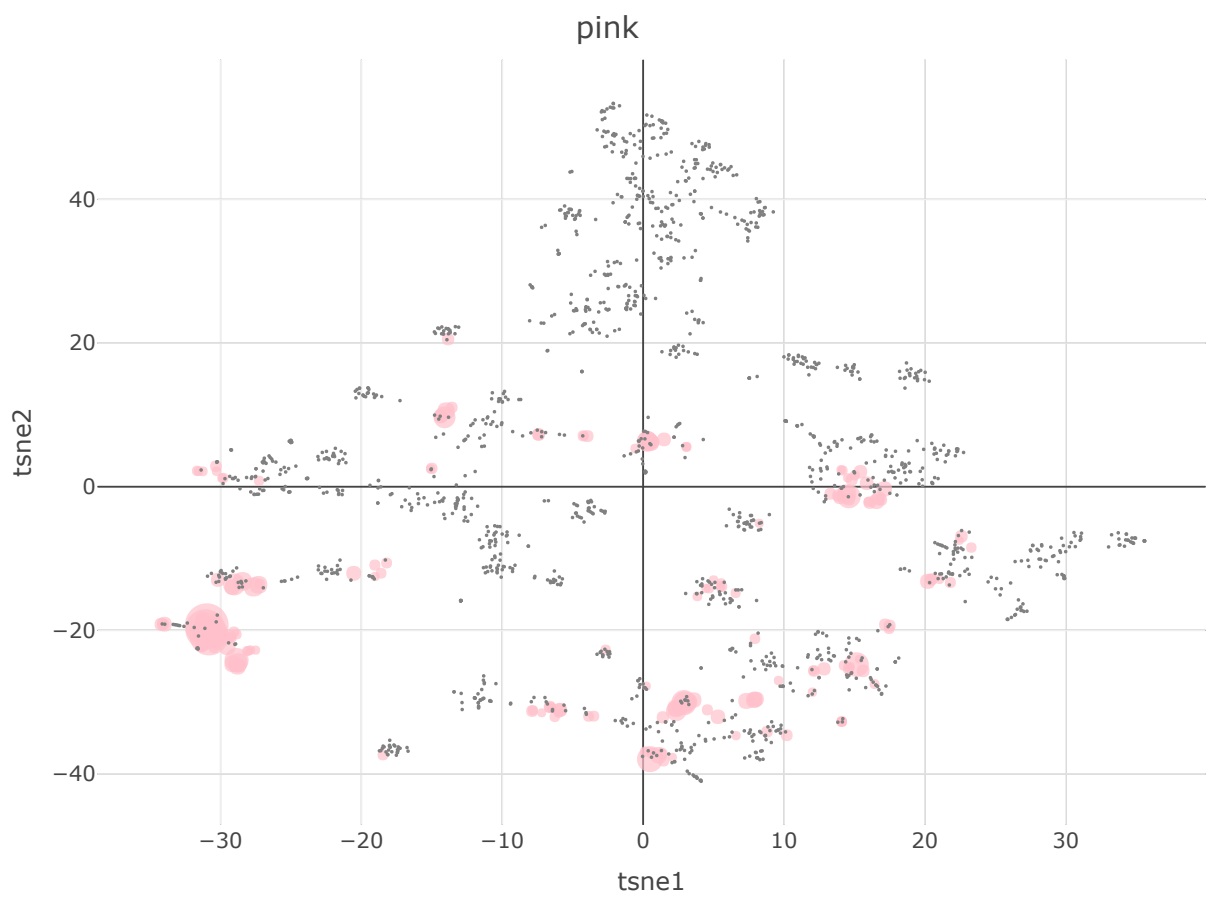

Supplement: S4 Fig — GO phenospace maps, as in Fig 3C, for all modules. (PDF) [file pcbi.1007343.s004.pdf]

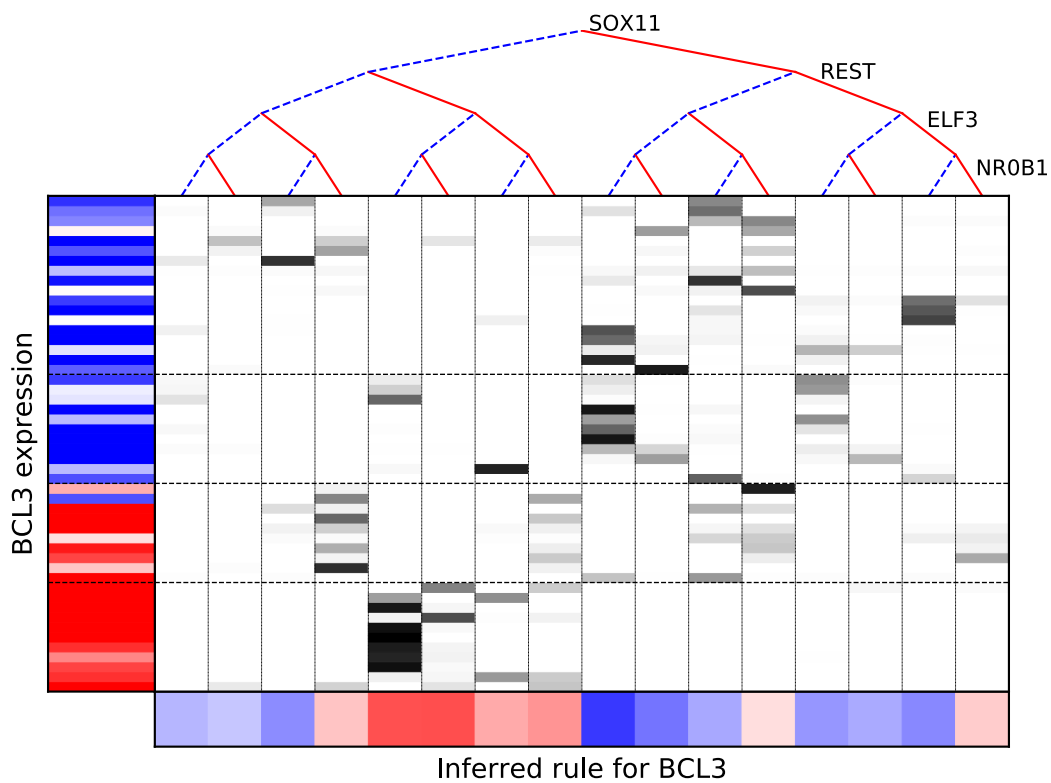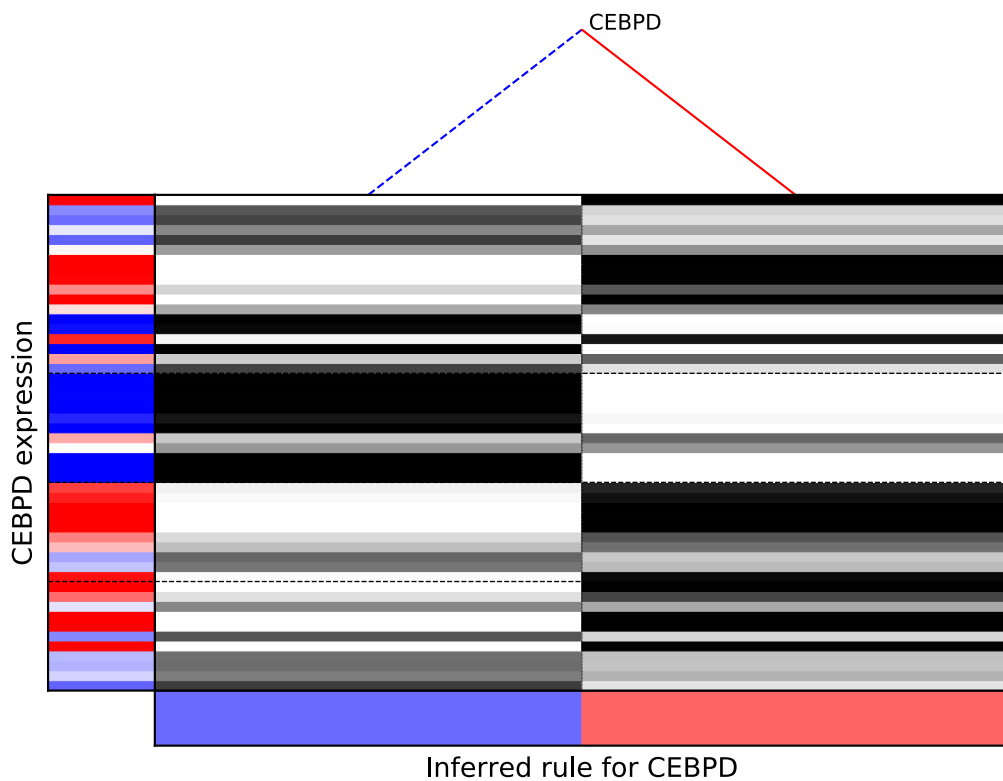

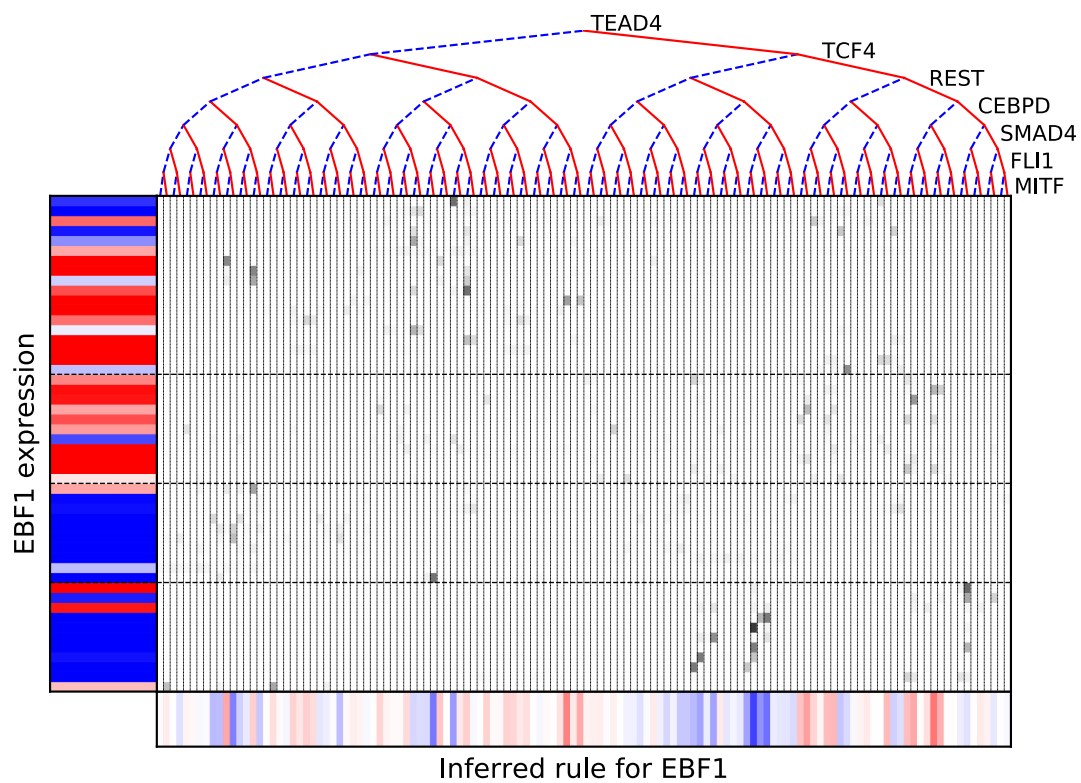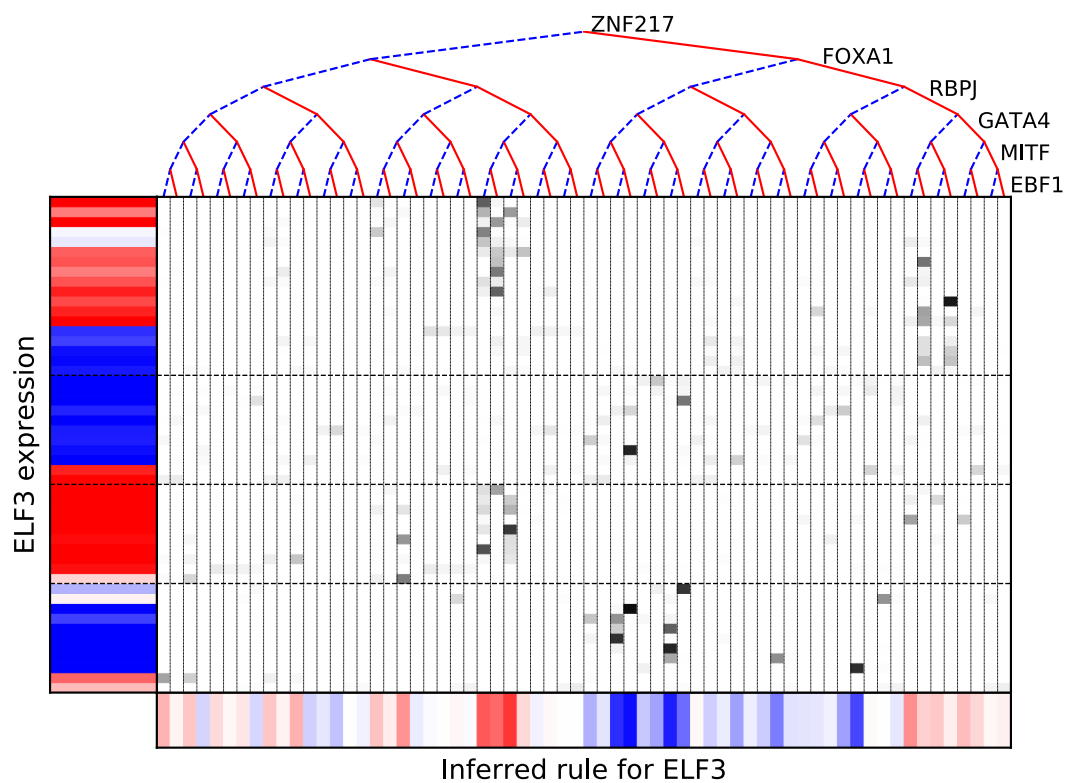

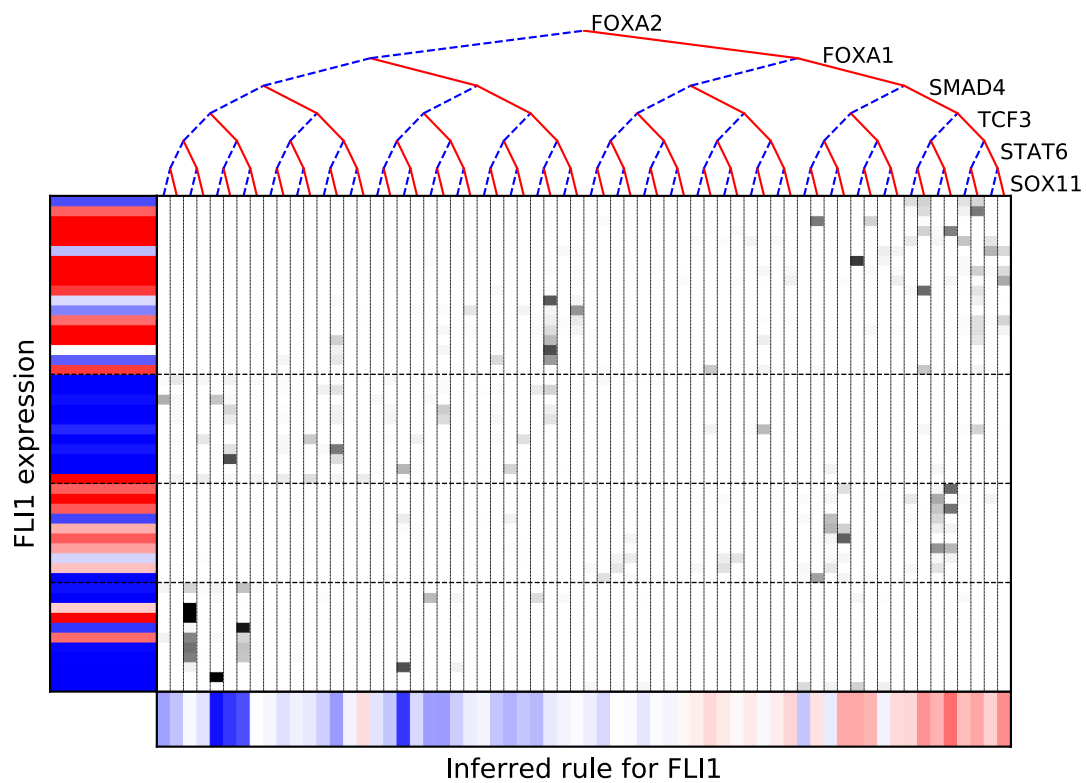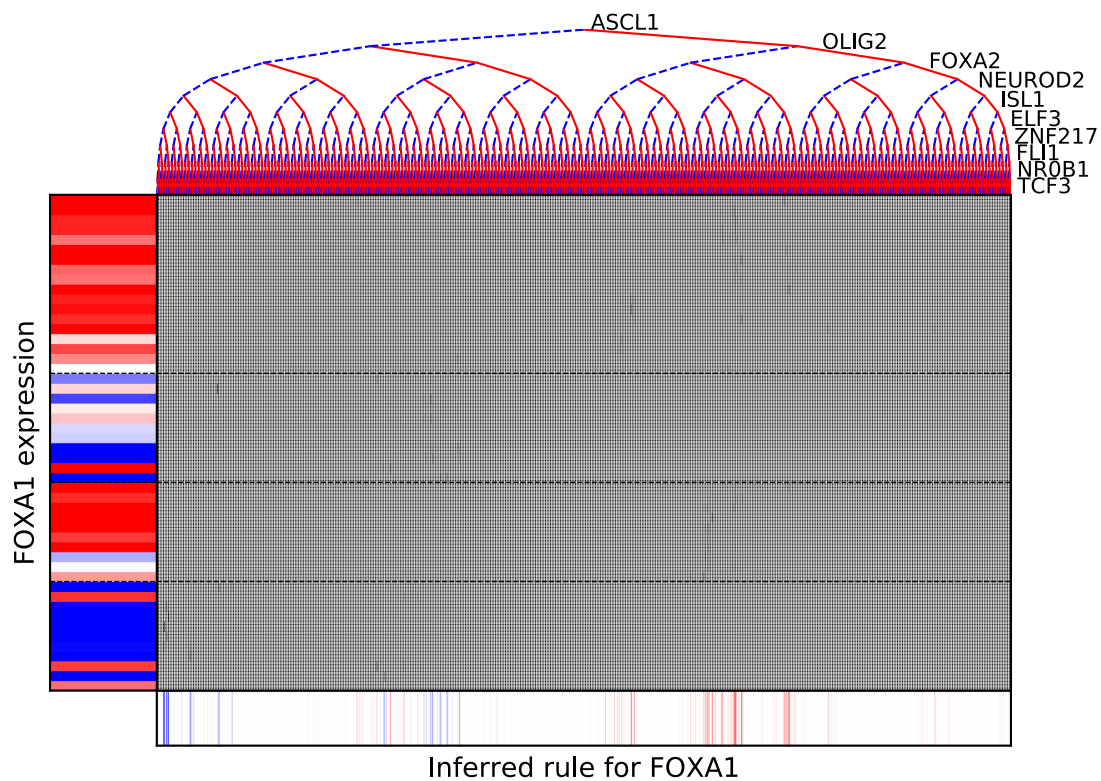

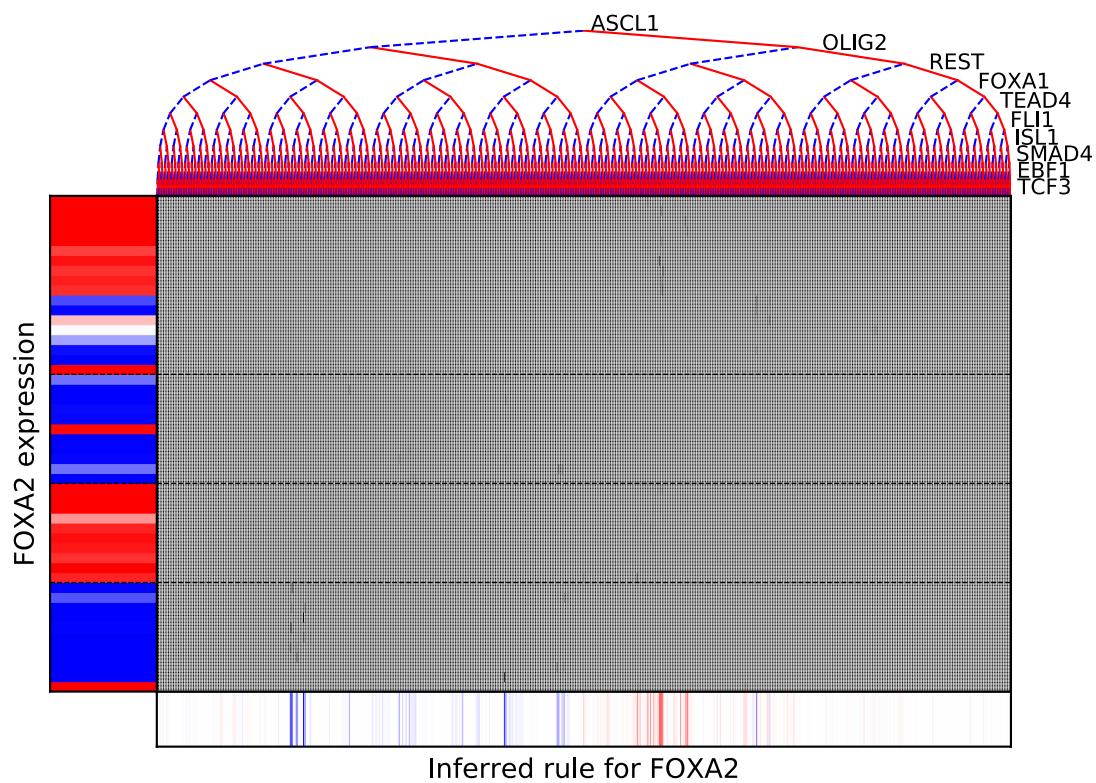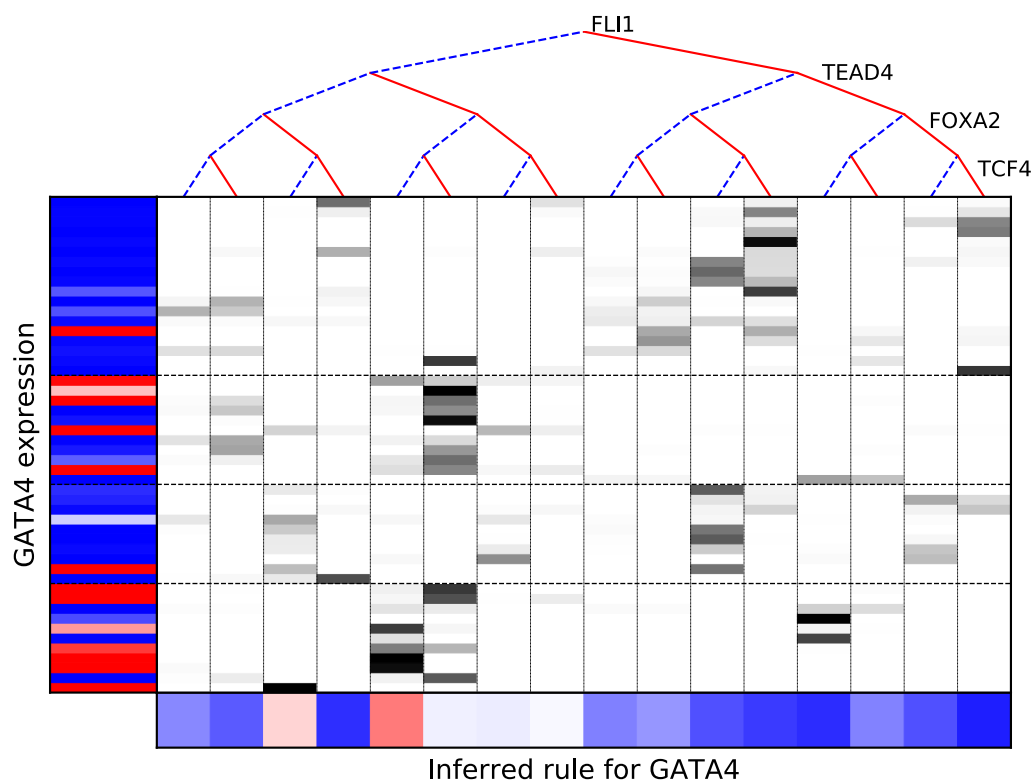

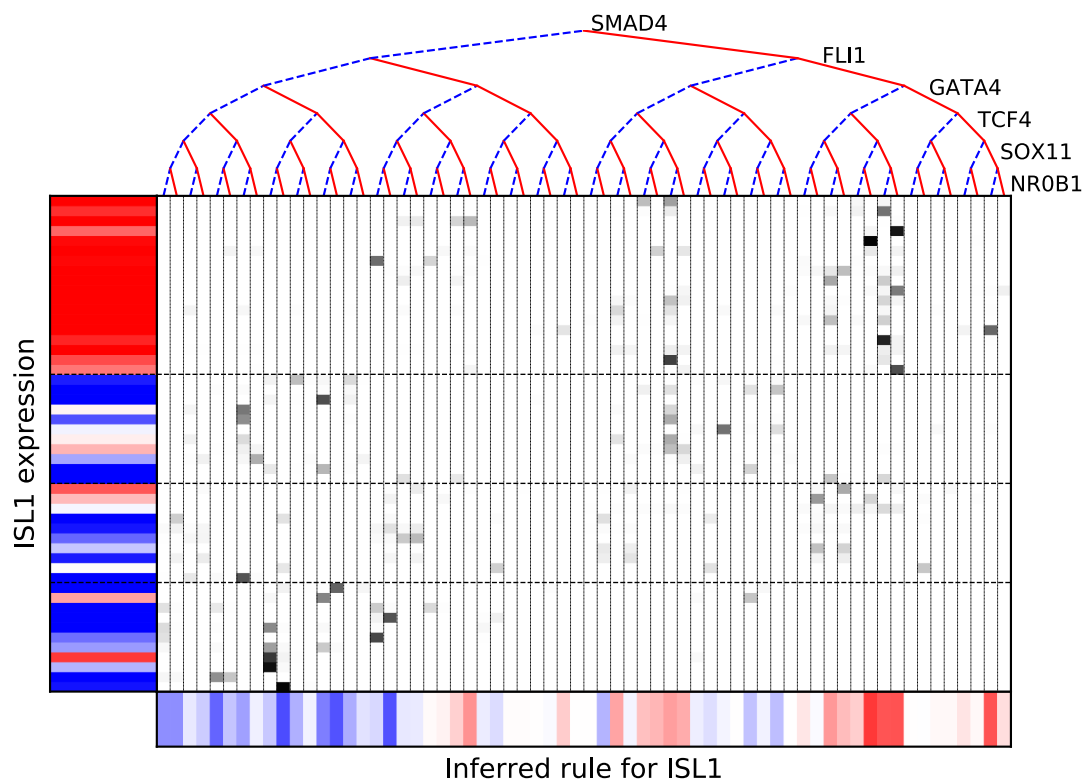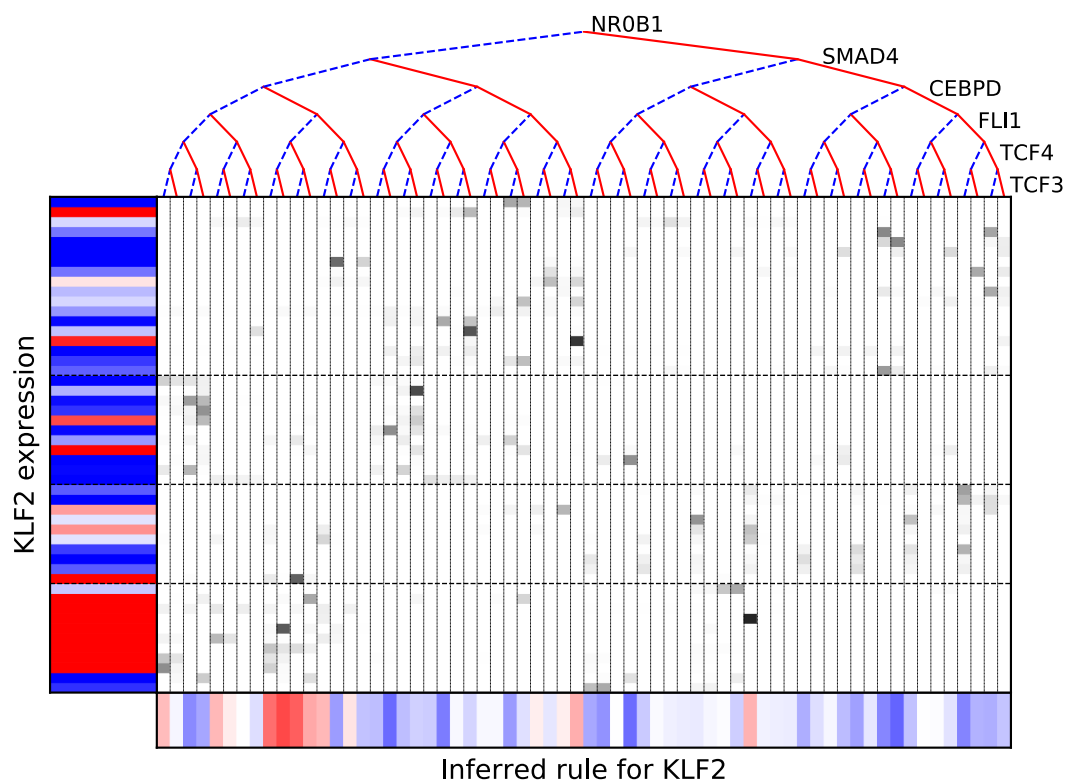

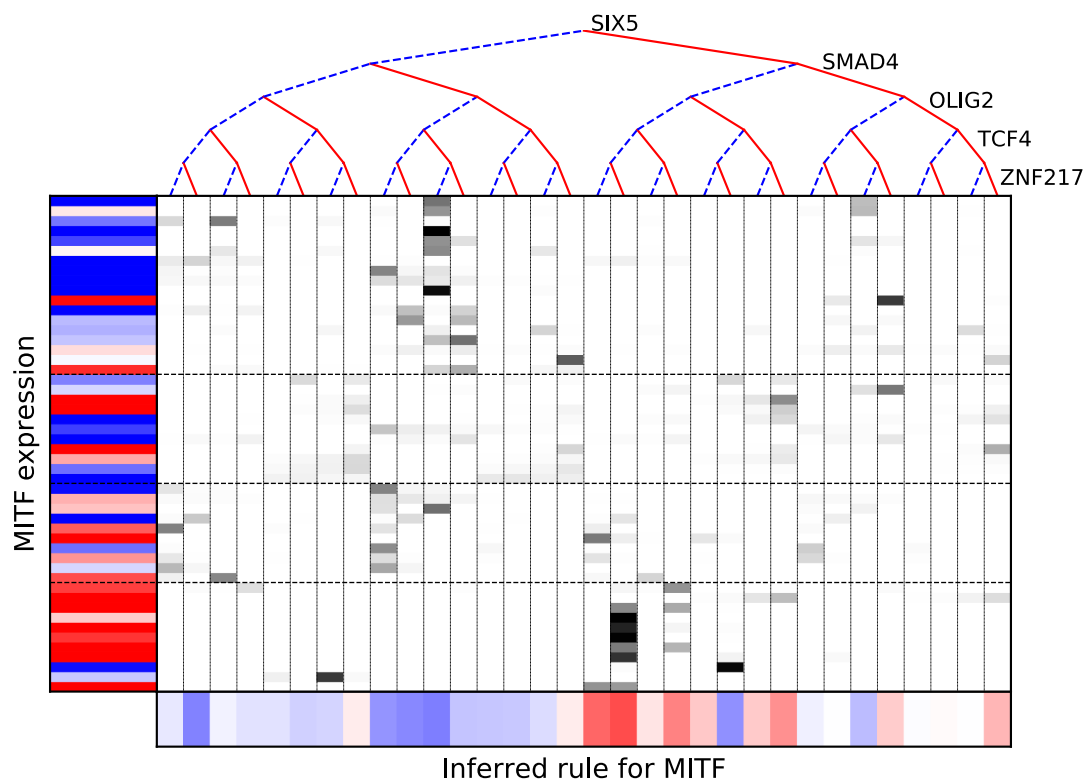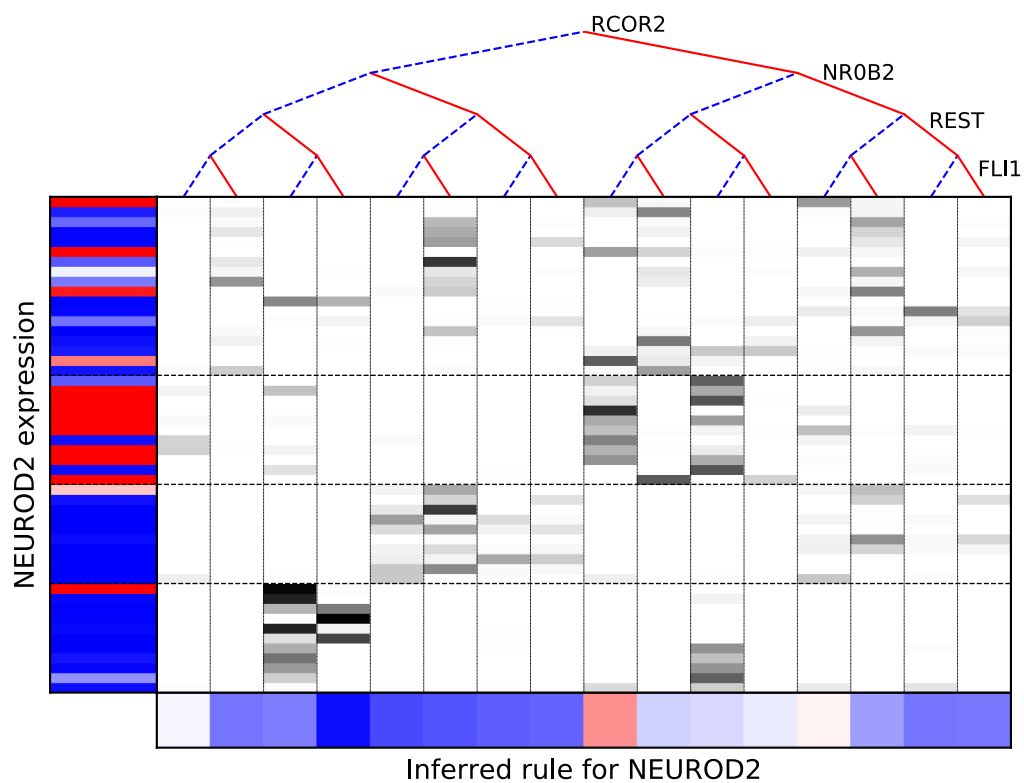

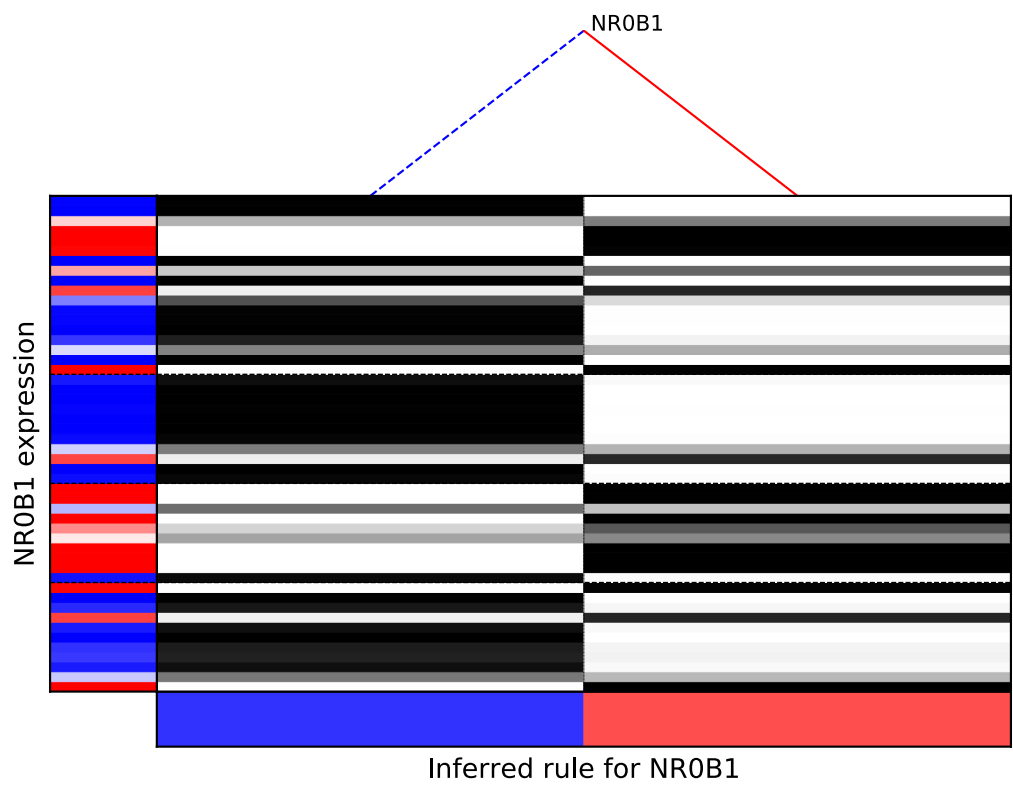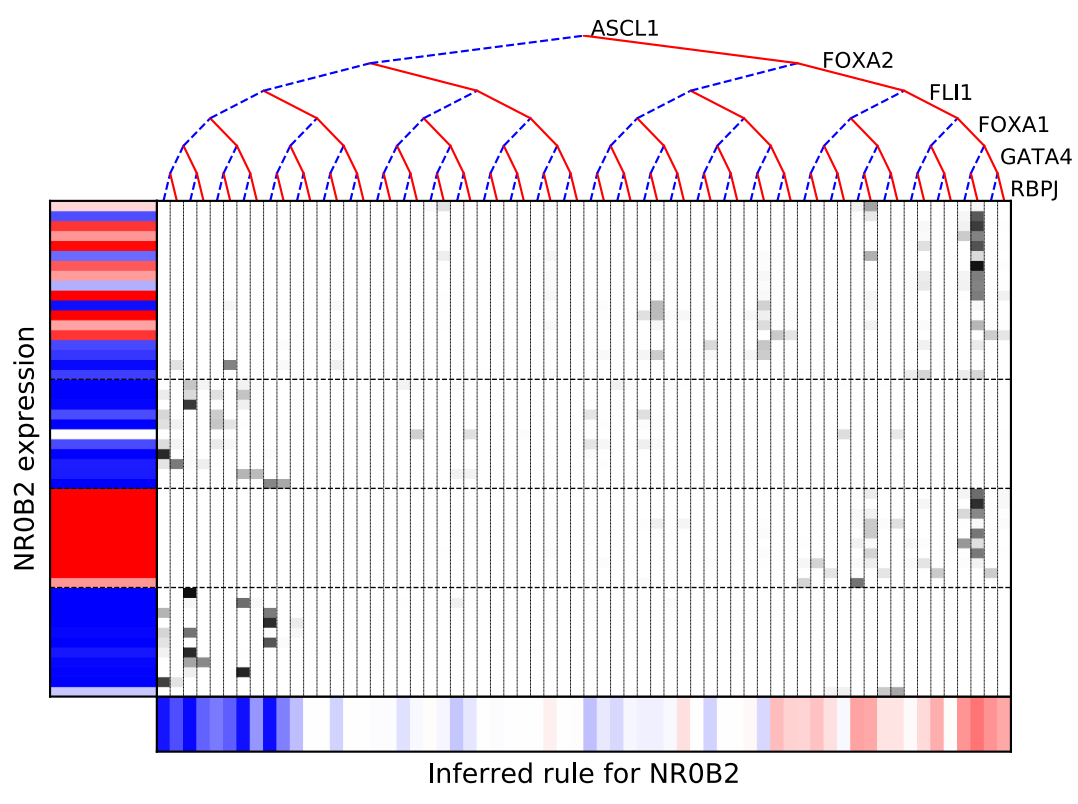

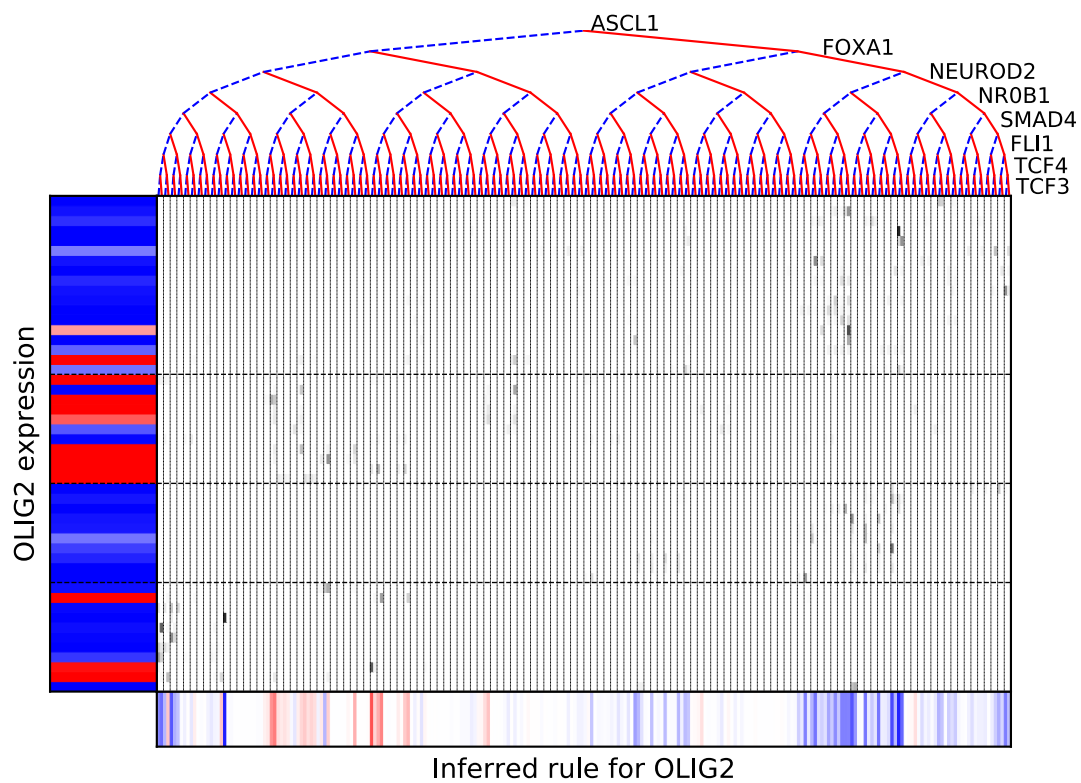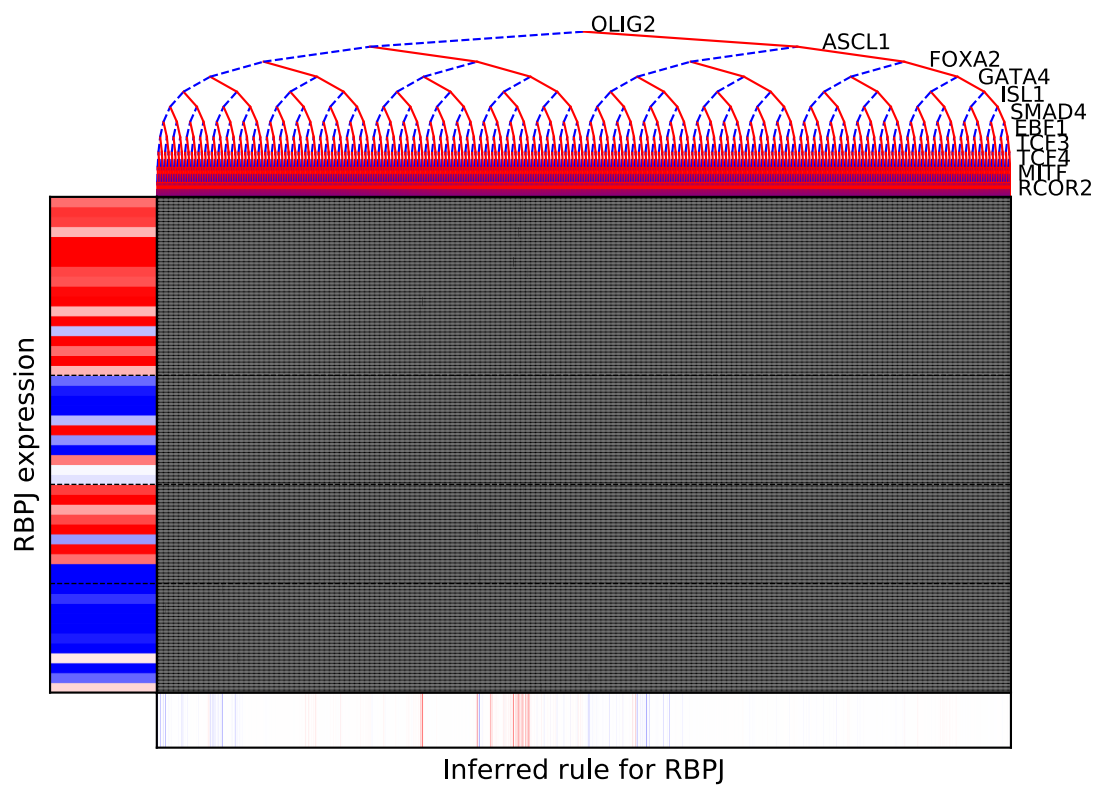

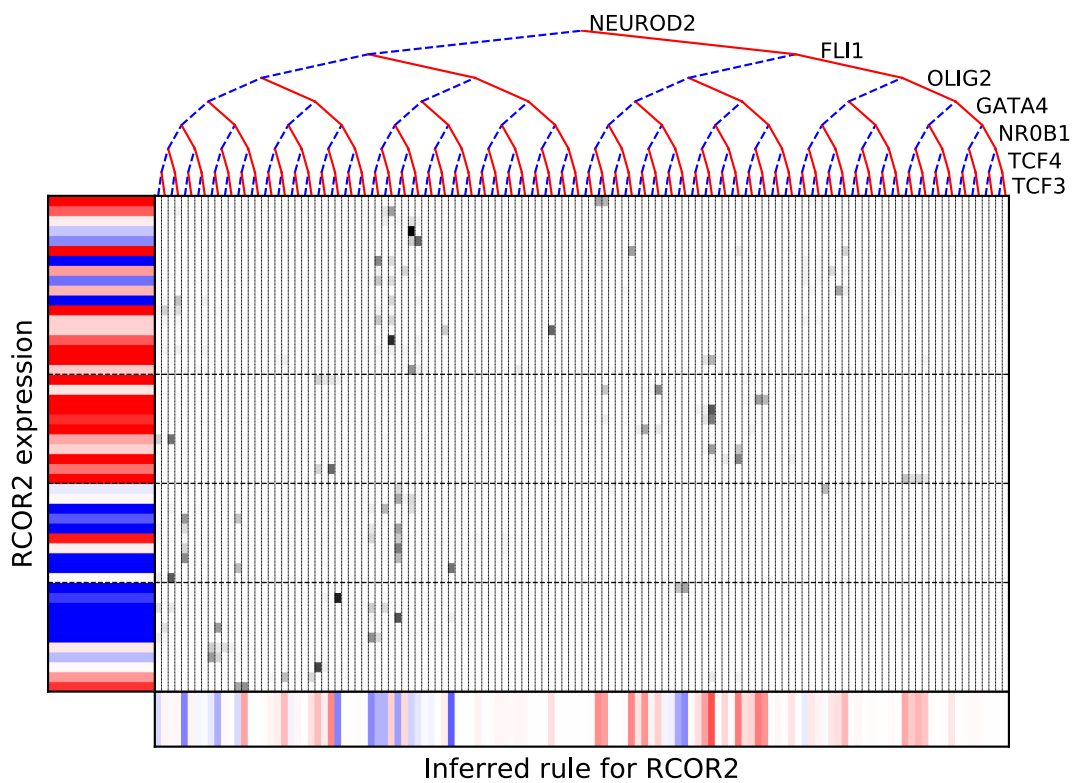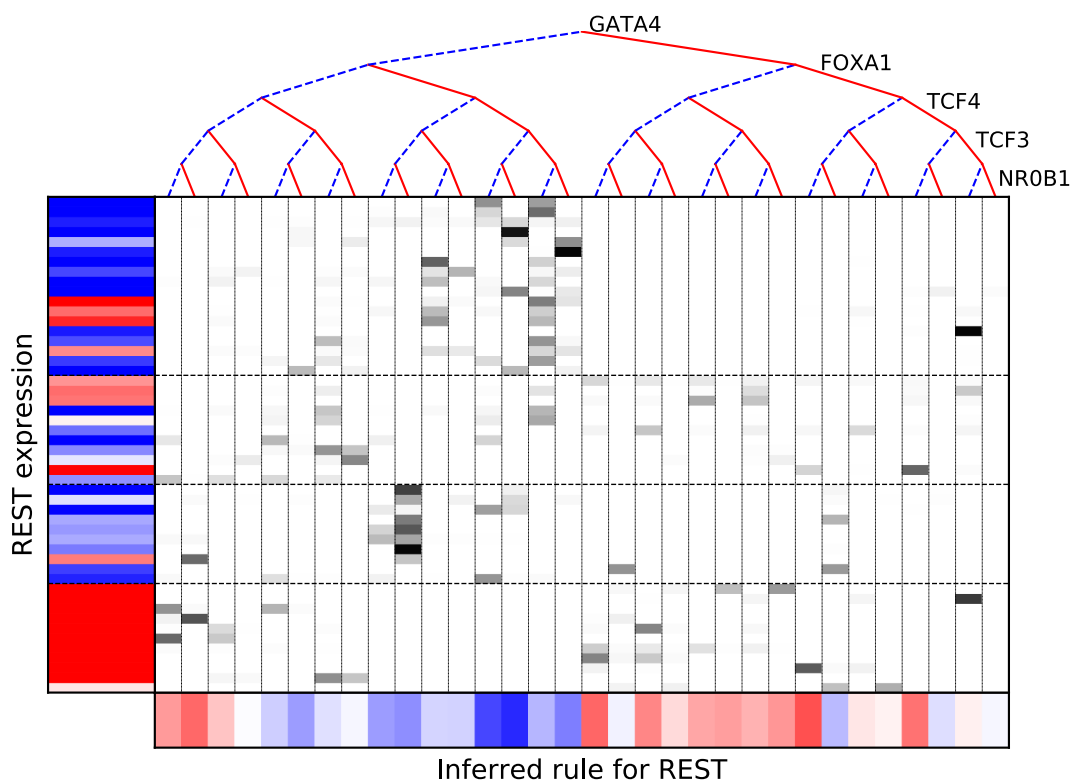

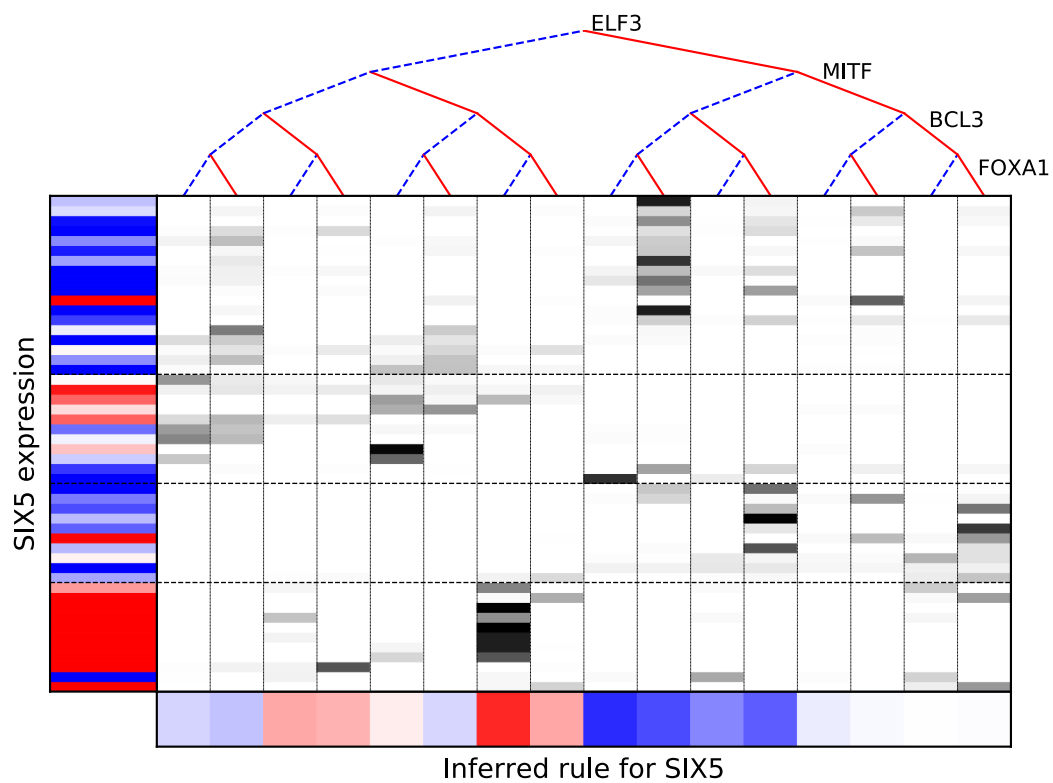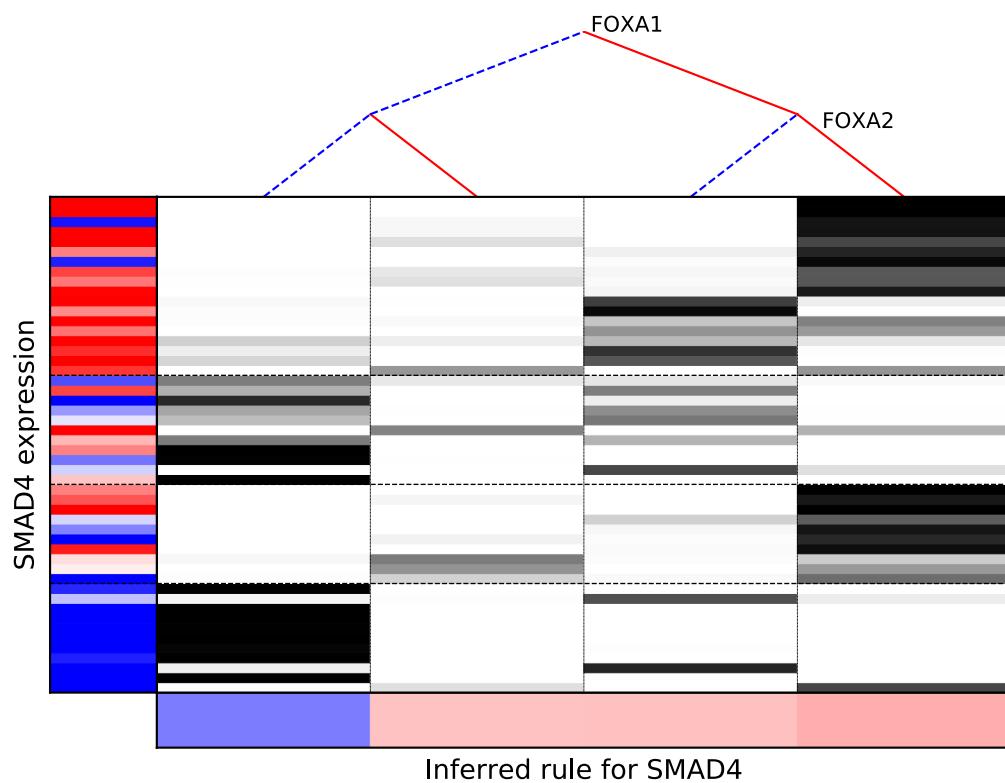

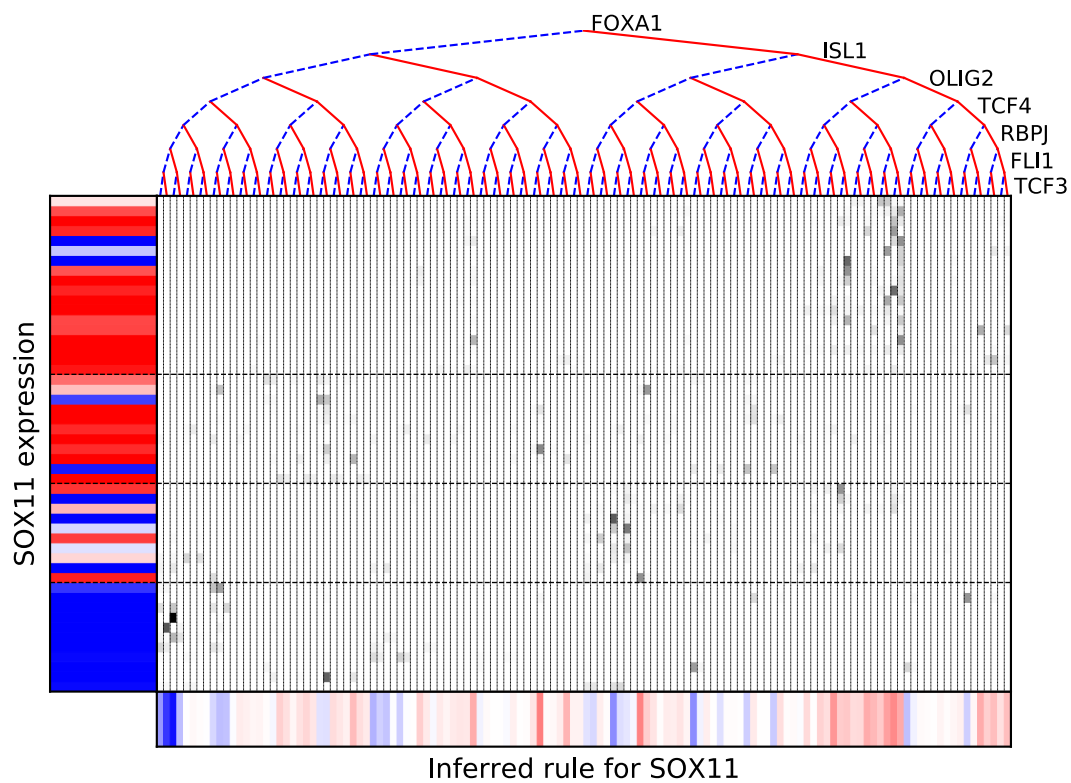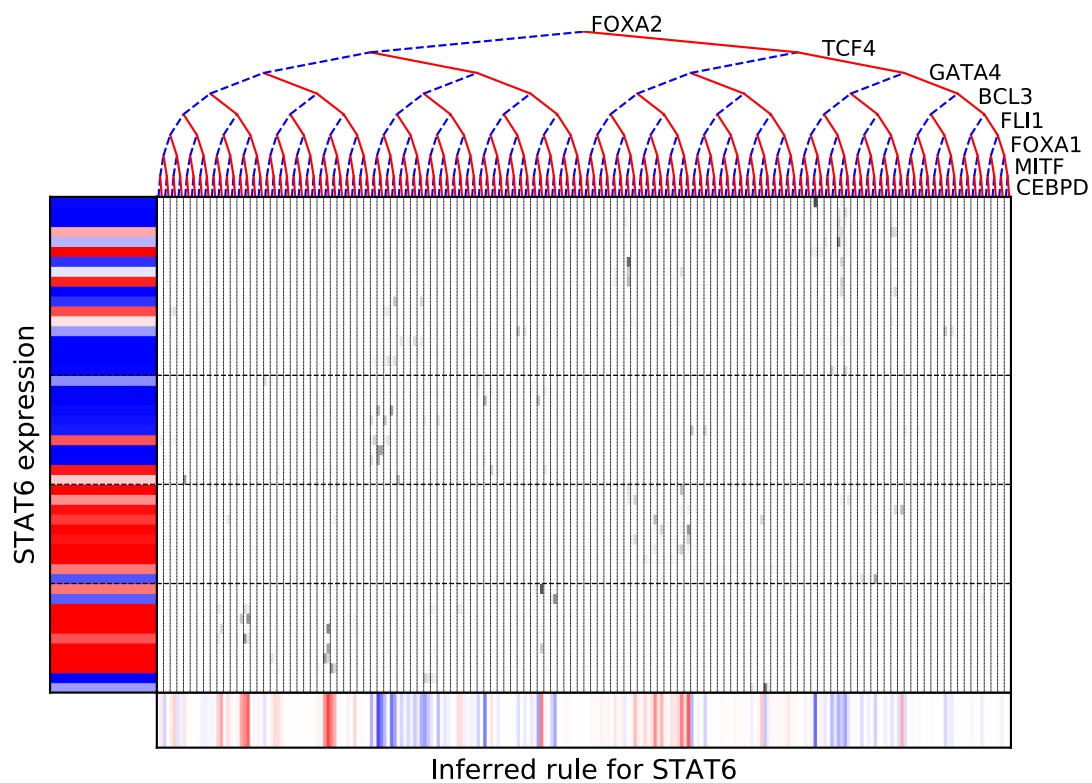

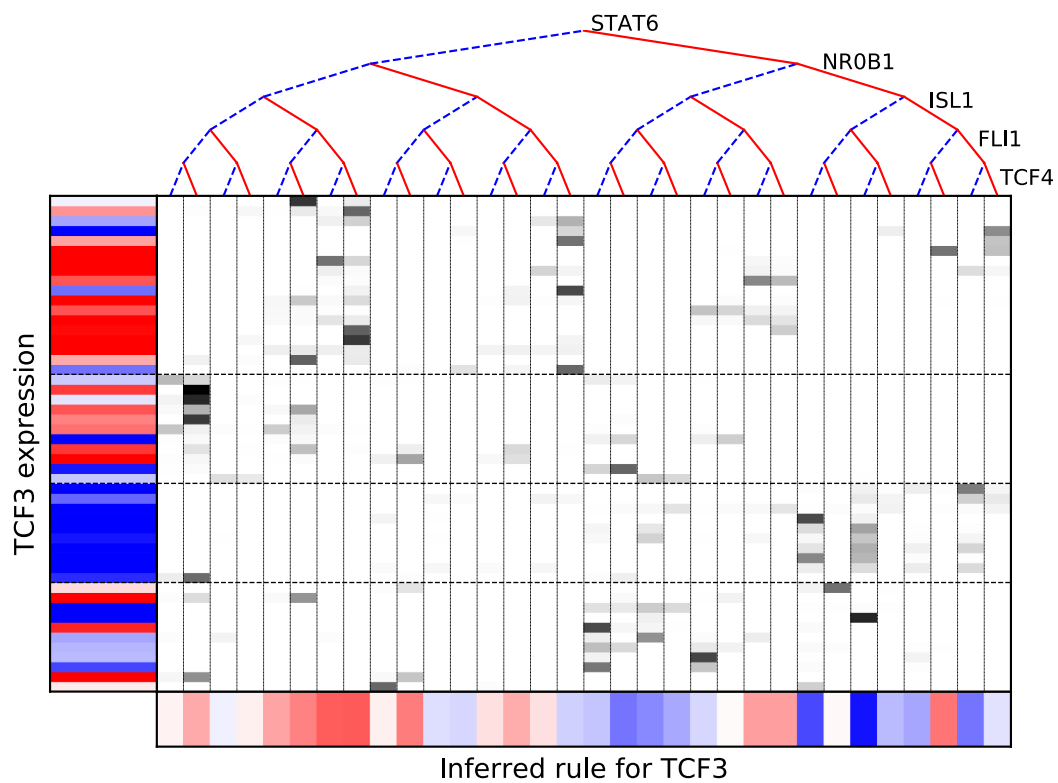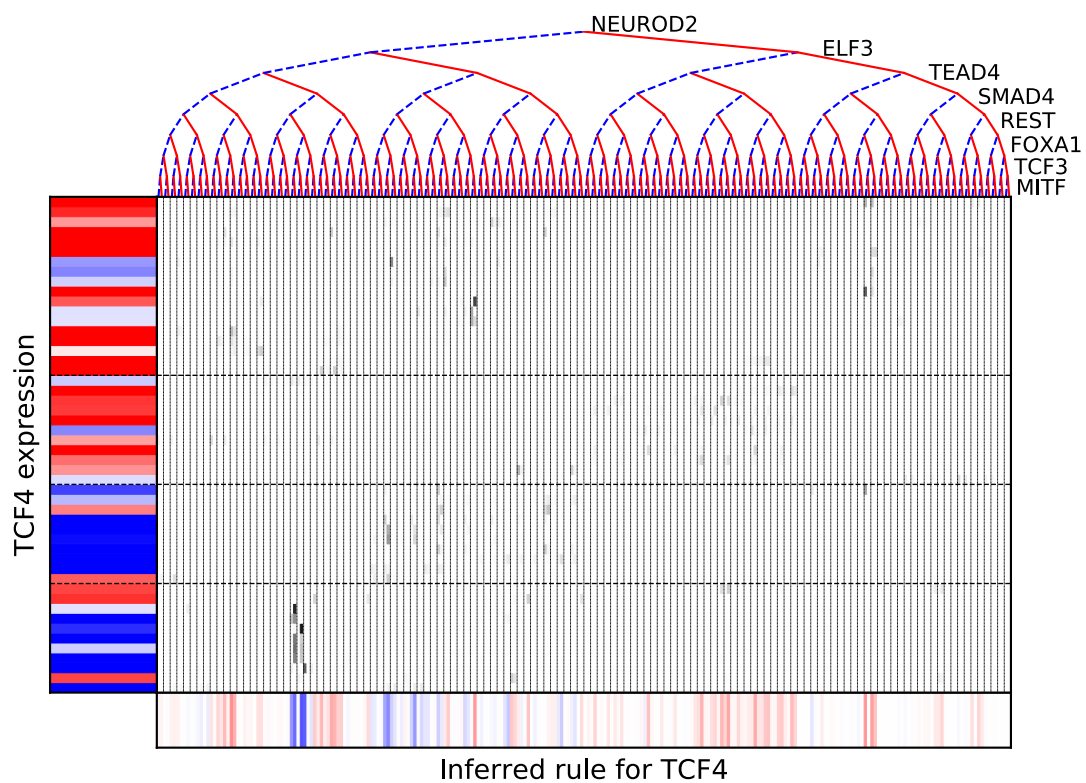

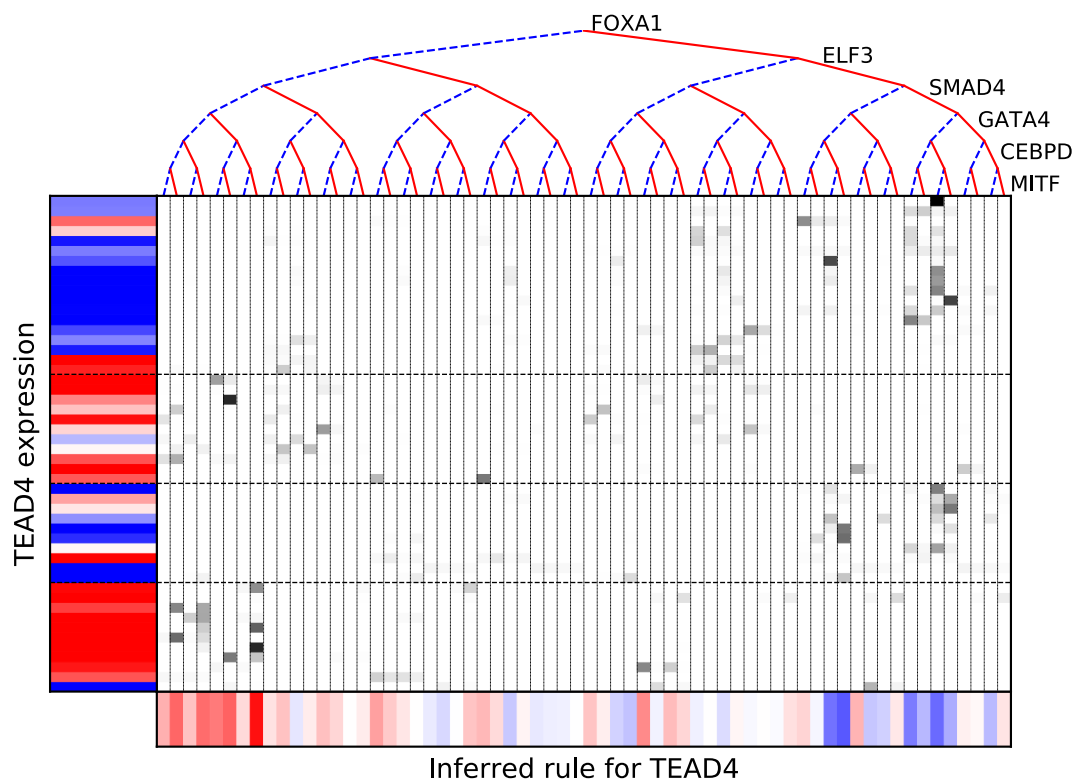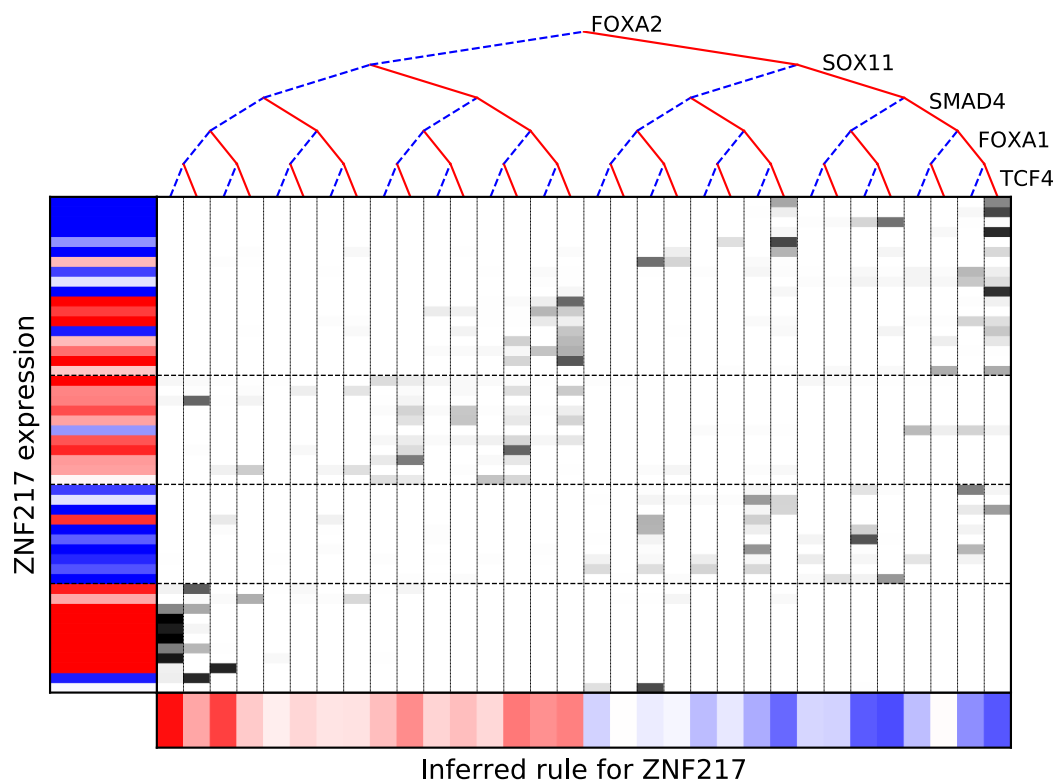

Supplement: S7 Fig — Rules for all TFs, as in Fig 7B. (PDF) [file pcbi.1007343.s007.pdf]
