# Supplementary material for: Systems-level network modeling of Small Cell Lung Cancer subtypes identifies master regulators and destabilizers
Source: PLoS Comput Biol. 2019 Oct 31;15(10):e1007343. doi: 10.1371/journal.pcbi.1007343 (PMC6860456; doi:10.1371/journal.pcbi.1007343)
Supplement: S1 File — (ZIP) [file pcbi.1007343.s009.zip › S1 Files/GO_map_plotly_overlay_black.html]

plotly


Search
